# An Unusual Presentation of Limited Granulomatosis with Polyangiitis Involving Vagina and Urethra

**DOI:** 10.1155/2017/9407675

**Published:** 2017-03-13

**Authors:** Sandra Soro Marín, Enrique Júdez Navarro, Manuela Sianes Fernández, Ginés Sánchez Nievas, Juan Gabriel Lorenzo Romero

**Affiliations:** ^1^Rheumatology Unit, Hospital General de Villarrobledo, Villarrobledo, Albacete, Spain; ^2^Department of Rheumatology, Complejo Hospitalario Universitario de Albacete, Albacete, Spain; ^3^Department of Urology, Complejo Hospitalario Universitario de Albacete, Albacete, Spain

## Abstract

Granulomatosis with polyangiitis (GPA) is a systemic necrotizing granulomatosis vasculitis characterized by predilection to affect small- and medium-sized blood vessels and commonly affects the upper and lower respiratory tract and kidneys in most cases. Genital involvement is reported in <1% of cases in large cohorts and nearly all cases have been in the setting of multisystemic disease or during the course of the disease in patients already diagnosed as GPA. A case is presented of uncommon limited urogenital GPA in a 66-year-old woman with an irregular mass occupying urethra and vagina. The patient showed a good response after Corticoids and Methotrexate.

## 1. Introduction

Granulomatosis with polyangiitis is a multisystemic vasculitis associated with anti-neutrophil cytoplasmic antibodies (ANCAs) characterized by necrotizing granulomatous inflammation of the upper and lower respiratory tract and kidney. Necrotizing vasculitis affects predominantly small-sized blood vessels [1].

Even though GPA is considered a systemic disease, limited forms may occur without evidence of systemic involvement (no vasculitic features) mainly affecting respiratory tract. Limited urogenital tract form is reported in <1% of cases. Gynaecological involvement is even much more unusual and occurs in a minority of patients. To the best of our knowledge, just one case of limited form with uterine cervix and vagina involvement has been reported in medical literature [2]. The diagnosis of GPA is mainly made by the histological demonstration of vasculitis, necrosis, and granulomatosis inflammation. Immunosuppressive agents, especially Cyclophosphamide, are the cornerstone of the treatment of GPA.

We report a case of a patient with a rare form of limited GPA affecting the urethra and vagina simultaneously as the first manifestation. Methotrexate was the treatment of choice for this limited GPA with a good response.

## 2. Case Report

**Figure 1:**
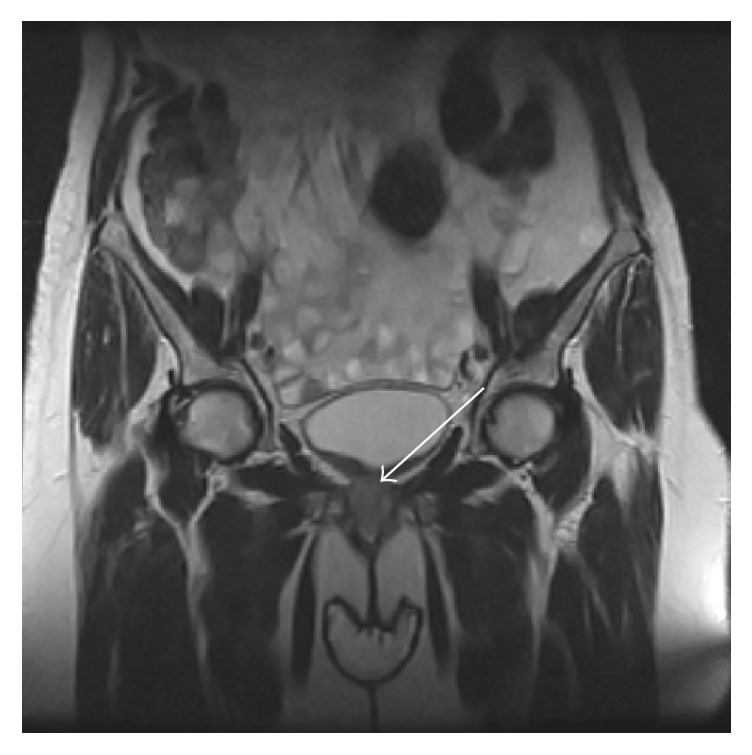
Coronal plane pelvic MRI showed an irregular mass with crenulated margins occupying the urethra and vagina.

**Figure 2:**
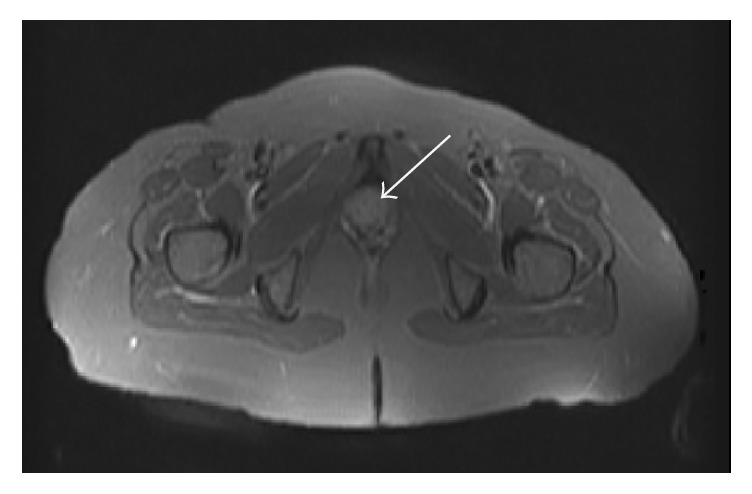
Axial plane pelvic MRI showed an irregular mass with crenulated margins occupying the urethra and vagina.

A 62-year-old woman presented with a 3-month history of vesical tenesmus without dysuria, vaginal bleeding, and low grade fever was referred to gynaecological and urological units. Her past medical history was significant for hypertension, osteoporosis, and B-thalassemia. She underwent hysterectomy and adnexectomy 12 years before due to fibroids. Clinical examination revealed abdominal pain and irregular hardening of anterior vagina encompassing urethra. No disorders involving respiratory tract, cardiovascular system, ear, or eyes were noted. A test of cervical smear and colposcopic evaluation were normal. Vital signs were normal, except temperature 37.5°C. Laboratory test revealed a hemoglobin level of 9,8 g/dL, 13430 leukocytes, and 630000 platelets. Urinalysis and intravenous urography were normal. Ultrasonography, abdomen computed tomography (CT), and pelvic magnetic resonance showed an irregular mass with crenulated margins occupying the urethra and vagina (Figures 1 and 2). Under the presumptive diagnosis of tumor, biopsy was carried with several specimens obtained. The biopsy showed a granulomatous inflammatory reaction, with small- and medium-sized vessels vasculitis and a hemorrhagic and necrotic background and without the characteristic features of caseating necrosis. The diagnosis of limited GPA was considered and patient was referred to Rheumatology Unit.

We proceeded to extend the study. Chest X-rays were normal and a skin test for tuberculosis was negative. Laboratory test revealed a sedimentation rate of 31 mm/h and C-reactive protein level of 20.7 mg/dL. The serum chemistry was unremarkable with creatinine of 1.0 mg/dL. Tumor markers were all in normal range. Anemia, thrombocytosis, and leukocytosis persisted. Anti-neutrophil cytoplasmic antibodies (c-ANCA) at a titer of 1/80 were noted. Rest of immunological studies was negative.

Prednisone (0,5/mg/kg/day) and oral Methotrexate (10 mg per week) were started with resolution of the mass and the symptoms. Sustained remission was achieved. Two years later, the patient was admitted to hospital for septic shock of urinary tract origin. Methotrexate was suspended and treatment with Prednisone (5 mg per day) was continued. The patient remained well; however two years later she began with a 2-week history of abdominal pain and vaginal bleeding. MRI showed mucosal edema at bladder neck and proximal urethra and persistence of vaginourethral fistula. Laboratory tests including ANCAs were normal. Prednisone dose was increased (0,5/mg/kg/day) and Azathioprine was started but had to be discontinued for hepatotoxicity. At this moment, five years after the onset of the symptoms, patient is in remission under low dose of Prednisone (2,5 mg per day), Methotrexate (7,5 mg per week), and Trimethoprim/Sulfamethoxazole three days per week for recurrent urinary tract infections prophylaxis.

## 3. Discussion

Granulomatosis with polyangiitis is an anti-neutrophil cytoplasmic antibodies- (ANCAs-) associated vasculitis characterized by granulomatous and necrotizing inflammation of small- and medium-sized arteries. It is an uncommon disease with a prevalence of approximately 22–157 cases per million and preferentially affects Caucasians in the 6th decade of life [3]. It is characterized by involving preferentially the upper and lower respiratory tract. Urologic and especially gynaecological manifestations of GPA are rare and only have been reported in <1% of cases with evident signs at this level [2, 4]. Information is derived from case reports and 3 small series [5–7]. In GPA, urogenital symptoms are mainly observed as part of generalized systemic disease. The diagnosis of limited urogenital GPA is uncommon [7].

Laboratory findings include elevated acute phase reactants, leukocytosis, thrombocytosis, and normocytic normochromic anemia. ANCAs are present in 75–87.5% of cases, of these 90% are directed against proteinase 3 (PR3) being highly specific for GPA. The remaining are against myeloperoxidase (MPO) [5–7]. Urogenital disease is an unusual presenting feature of GPA occurring in 12–18% of patients [5, 6]. Most of these patients will develop systemic disease at some time during the course of the illness and only few cases will remain as urogenital limited GPA. The case presented did not developed systemic manifestations and the main symptom was urogenital. Recurrences are observed in 36–50% of patients with urological manifestations [5–7]. Furthermore, some cases are asymptomatic or with mild manifestations because genital examination is not a routine in the GPA management [8].

To The best of our knowledge there are 19 published cases of GPA presenting as an inflammatory lesion involving urogenital tract which are discovered in imaging studies and are described like pseudotumors [2, 9–13].

The most common urogenital manifestations are prostatitis (the urological manifestation with the highest number of reported cases, approximately 40), penile necrosis (20 cases reported) [7], orchitis (12.5–36% GPA patients with urogenital disease), ureter involvement (20 reports), renal masses (18 cases), urethritis (8 cases), and epididymitis (4 patients) [3]. Vagina or cervix is very rarely affected (1 case) [2].

There are no controlled studies about the therapeutic approach of urogenital GPA. Therefore, general GPA treatment can be applied to the urogenital involvement. Treatment will depend on the severity of the symptoms [14]. In the extensive disease, Glucocorticoids, Cyclophosphamide, or Rituximab will be considered. Methotrexate or Azathioprine is valid options in limited disease [15]. Most patients have an excellent response with immunosuppressive therapy. Surgical treatment is reserved for temporary measure to relief obstructive symptoms.

## 4. Conclusion

We describe a rare case of GPA with isolated urogenital involvement as the only manifestation, without systemic symptoms, where diagnosis was provided by a mass biopsy and supported by analytical findings. We would like to stress on the importance of a genital examination in patients with systemic illness. Immunosuppressive drugs should be used as first-line therapy to avoid unnecessary surgery and prevent recurrences.
